# Nepal’s worst dengue outbreak is a wake-up call for action

**DOI:** 10.1093/jtm/taad112

**Published:** 2023-08-16

**Authors:** Sanjeev M Bijukchhe, Matilda Hill, Bipin Adhikari, Ashis Shrestha, Shrijana Shrestha

**Affiliations:** Department of Paediatrics, Patan Academy of Health Sciences, Kathmandu, Nepal; Department of Paediatrics, University of Oxford, Oxford, UK; Department of Paediatrics, University of Oxford, Oxford, UK; Mahidol-Oxford Tropical Medicine Research Unit, Faculty of Tropical Medicine, Mahidol University, Bangkok, Thailand; Centre for Tropical Medicine and Global Health, Nuffield Department of Medicine, University of Oxford, Oxford, UK; Department of Paediatrics, Patan Academy of Health Sciences, Kathmandu, Nepal; Department of Paediatrics, Patan Academy of Health Sciences, Kathmandu, Nepal

## Abstract

Nepal had its worst dengue outbreak in 2022. Climate change, urbanization and increased transportation networks have favoured dengue mosquito vectors. As the monsoon season approaches, dengue outbreak seems inevitable. Strengthening the national dengue preventive strategy, healthcare planning and resource allocation is essential to avoid future outbreaks.

In 2022, Nepal faced its worst ever outbreak of dengue, with over 50 000 cases and >50 deaths reported across all 77 districts, with the highest number of cases in the capital city, Kathmandu.^1^ Dengue is becoming increasingly prevalent at higher altitudes such as Kathmandu (altitude 1400 m) and has been attributed to rising temperatures (climatic changes) creating a conducive environment for Aedes mosquito breeding.^2^ However, this number likely underestimated the true burden of the disease, as up to 80% of dengue infections can be mild or asymptomatic.^3^ The epidemiological analysis of national data has shown an escalating trend over the years and worst is imminent as monsoon season looms closer.

The outbreak led to a surge in emergency department attendances at Patan hospital, where over 6600 cases of confirmed dengue were treated, and 576 patients required hospital admission between 1 August 2022 and 29 November 2022. At the peak of the outbreak, emergency deparment (ED) attendances doubled, placing tremendous pressure on the hospital and resulting in delays in diagnostics and treatment for both dengue and non-dengue patients. The hospital implemented various measures, including streaming of potential dengue cases into a separate area of ED, a hospital-specific guideline detailing the investigation, management and admission criteria for dengue was devised and training for staff was provided in the form of a video which could be accessed remotely and flexibily around clinical shifts.

The scale of the outbreak in Nepal was unprecedented; however, it was not unforeseen.^4^ Dengue cases had been increasing since the disease was first identified in Nepal in 2004, with the previous largest outbreak occurring in 2019 when 17 992 cases were reported.^5^ All four serotypes of dengue now circulate in Nepal. Climate change, urbanization and increasing transportation networks with other endemic regions have created increasingly favourable conditions for the mosquito vectors of dengue, *Aedes aegypti* and *Aedes albopictus*, and for the transmission of the disease in Nepal.^4^ This pattern is reflected globally; dengue is now the most prevalent mosquito-borne viral disease, causing an estimated 390 million infections in over 100 countries annually.^3^

The government response in Nepal, coordinated by the Ministry of Health & Population’s Epidemiology and Disease Control Division (EDCD) and supported by the World Health Organization (WHO), included dengue information sharing with all administrative levels across Nepal, provision of rapid diagnostic tests (RDTs) and initiation of vector surveillance and ‘search and destroy’ mosquito campaigns in the most affected districts of Kathmandu and Lalitpur.^1^ Currently, the Dengue surveillance is being highly prioritized by the EDCD of Nepal and are utilizing RDTs, which are readily accessible even at the community level. However, there could be misdiagnosis/underdiagnosis of mild and moderate cases of dengue fever and more so among individuals seeking treatment from private healthcare services. However, diagnostic capabilities were limited; diagnoses were dependent on RDTs which could not differentiate between different dengue serotypes. All three serotypes of the virus was detected in the outbreak.

Dengue outbreak is an annual problem in Nepal coinciding with the monsoon season. Without strengthening of the existing national dengue prevention strategy, healthcare planning and further resource allocation, dengue outbreaks in Nepal is likely to continue to occur on a larger and more devastating scale. Immunity to dengue is serotype-specific; individuals infected during this outbreak will not be protected against other serotypes, and indeed there is evidence to suggest that they may be at increased risk of severe disease if infected with heterologous serotypes during future outbreaks.^4^ This could overwhelm the healthcare system and result in significant mortality.

While there is a new vaccine in the pipeline, this is not a solution to the short-term threat of dengue outbreaks in Nepal. The TAK-003 vaccine is now approved for use in Indonesia and has recently been endorsed by the European Medicines Agency after results showing good overall efficacy of 70.8% against hospitalized dengue at 3 years.^5^^,^^6^ Ongoing evaluation will be critical in determining whether the TAK-003 vaccine will be a viable option for Nepal.

A multi-faceted approach is required to limit the immediate risks presented by future outbreaks. Firstly, improved vector control methods must be implemented nationally with rigorous monitoring and evaluation. Eliminating potential breeding sites for Aedes mosquitoes requires an increased focus on removing stagnant water collections in all possible locations such as utensils, bottles, drums, drains, roads, pots, tyres and ponds.^7^ Biological strategies such as *Wolbachia*, an intracellular bacterium which limits vector proliferation and lifespan, interfering with dengue transmission, should be considered as an environmentally and financially sustainable option in mosquito-dense urban areas, based on strong supportive evidence from trials in Indonesia and Brazil.^7–9^Secondly, public health campaigns must be strengthened; awareness of dengue fever and understanding of prevention measures are currently very low.^10^ Community-level interventions to eliminate potential habitats for mosquitoes, and strategies to prevent mosquito bites such as use of insecticide repellents should be promoted. Thirdly, surveillance and diagnostic capacity must be improved to enable early detection of outbreaks and identification of responsible serotypes. Finally, further investment must be made in the training of healthcare workers to identify and manage severe dengue; interventions to support staff such as those introduced at Patan hospital should be widely adopted.

As climate change continues to cause temperatures to rise in Nepal, enabling *Aedes* mosquitoes to live at ever-higher altitudes, it is paramount that strategies to limit future dengue outbreaks are implemented across the country.
